# Calcium Involved in the Enrichment of γ-Aminobutyric Acid (GABA) in Broccoli Sprouts under Fructose Treatment

**DOI:** 10.3390/plants12020224

**Published:** 2023-01-04

**Authors:** Qinling Wei, Keqin Xie, Hongfei Wang, Xingfeng Shao, Yingying Wei, Yi Chen, Shu Jiang, Mengze Cao, Jisuan Chen, Feng Xu

**Affiliations:** 1 Zhejiang-Malaysia Joint Research Laboratory for Agricultural Product Processing and Nutrition, College of Food and Pharmaceutical Sciences, Ningbo University, Ningbo 315211, China; 2 Seymour College, Glen Osmond, SA 5064, Australia; 3 Haitong Food Group Co., Ltd., Ningbo 315100, China

**Keywords:** broccoli sprouts, γ-aminobutyric acid (GABA), fructose treatment, glutamate decarboxylase (GAD), calcium

## Abstract

The effect of fructose on γ-aminobutyric acid (GABA) content and its metabolic pathway in broccoli sprouts was investigated. The results demonstrated that the fructose treatment not only significantly increased the fresh weight, GABA, and glutamate contents in sprouts, but also promoted the activity of glutamic acid decarboxylase (GAD) and the expressions of *BoGAD1* and *BoGAD2*. Meanwhile, fructose treatment inhibited the stem length of broccoli sprouts and enhanced the abscisic acid (ABA) production in comparison with the control. Ca^2+^, CaM contents, and *BoCaM2* expression in broccoli sprouts were also stimulated after fructose treatment. Exogenous fructose increased inositol trisphosphate (IP_3_) content and activated the activity of phosphatidylinositol-specific phospholipase C (PI-PLC) and the expression of *BoPLC2*, contributing to Ca^2+^ influx into the cells. These results suggested that Ca^2+^ played an essential role in GABA enrichment under fructose treatment, which may be associated with GAD and PI-PLC.

## 1. Introduction

γ-aminobutyric acid (GABA) is a non-protein amino acid and is widely distributed in plants, animals, and bacteria [1]. It is an important inhibitory neurotransmitter in the human brain [2], antidepressant [3], and improver of insomnia effects [4]. In plants, GABA acts as a factor of resistance to adversity [5]. GABA is mostly produced from glutamate (Glu) and is catalyzed by glutamic acid decarboxylase (GAD) [6]. About 80% of GABA formed in tea leaves under anoxia is produced through GAD catalysis [7].

Calcium plays a key role in signal transduction in plant cells. GAD in plants is a CaM-binding protein that can be activated by Ca^2+^/CaM, thereby increasing GABA content [8]. Exogenous calcium chloride (CaCl_2_) could promote GABA accumulation in shredded carrots [9]. Similarly, CaCl_2_ activated GABA shunt through extracellular Ca^2+^ influx, which enhanced the GABA production of fresh-cut pears [10].

Broccoli sprouts are gaining interest for their bioactive compounds such as glucosinolates. Broccoli sprouts contain dozens of times more glucosinolates than broccoli florets [11]. Great efforts have been made to enhance glucoraphanin content and health-promoting metabolites in broccoli sprouts [12,13]. The accumulation of active compounds was induced by treating broccoli sprouts with slightly acidic electrolyzed water [14]. CaCl_2_-HCl electrolyzed water treatment enriched the amount of glucoraphanin and calcium contents in broccoli sprouts [15]. In our previous study, mannose treatment significantly increased the production of GABA in broccoli sprouts [16]. However, little focus has been given to the relationship between GABA metabolism and calcium in broccoli sprouts. Therefore, the effect and detailed mechanism regulating GABA accumulation under fructose treatment in broccoli sprout germination was elucidated. 

## 2. Results

### 2.1. Effect of Fructose Treatment on Growth of Broccoli Sprouts

**Figure 1:**
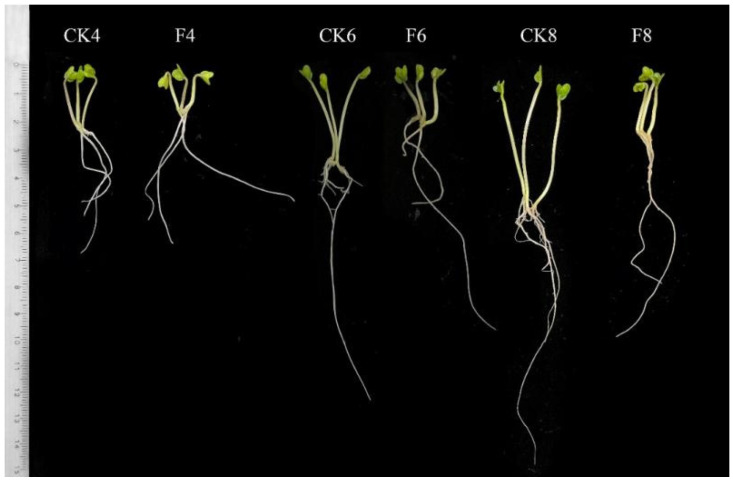
Effect of fructose treatment on growth performance of broccoli sprouts. The photograph of broccoli sprouts was taken 4, 6, and 8 days after germination. Broccoli seeds (Brassica oleracea var. italica cv. Yanxiu) were bought from Sakata Seed (Yokohama, Japan) Corporation.

**Figure 2:**
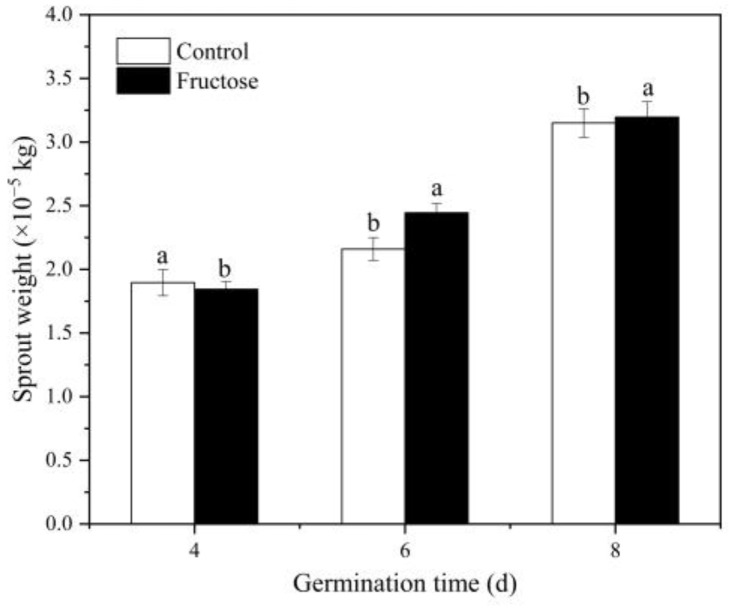
Effect of fructose treatment on weight of broccoli sprouts. Vertical bars represent the mean ± standard errors. Data with different letters are signed significantly at *p* < 0.05.

The results presented in Figure 1 showed that the stem length of the sprouts was inhibited under fructose treatment, while the weight of the sprouts increased (Figure 2).

### 2.2. Fructose Treatment Increased the Contents of GABA and Glutamate in Broccoli Sprouts

**Figure 3:**
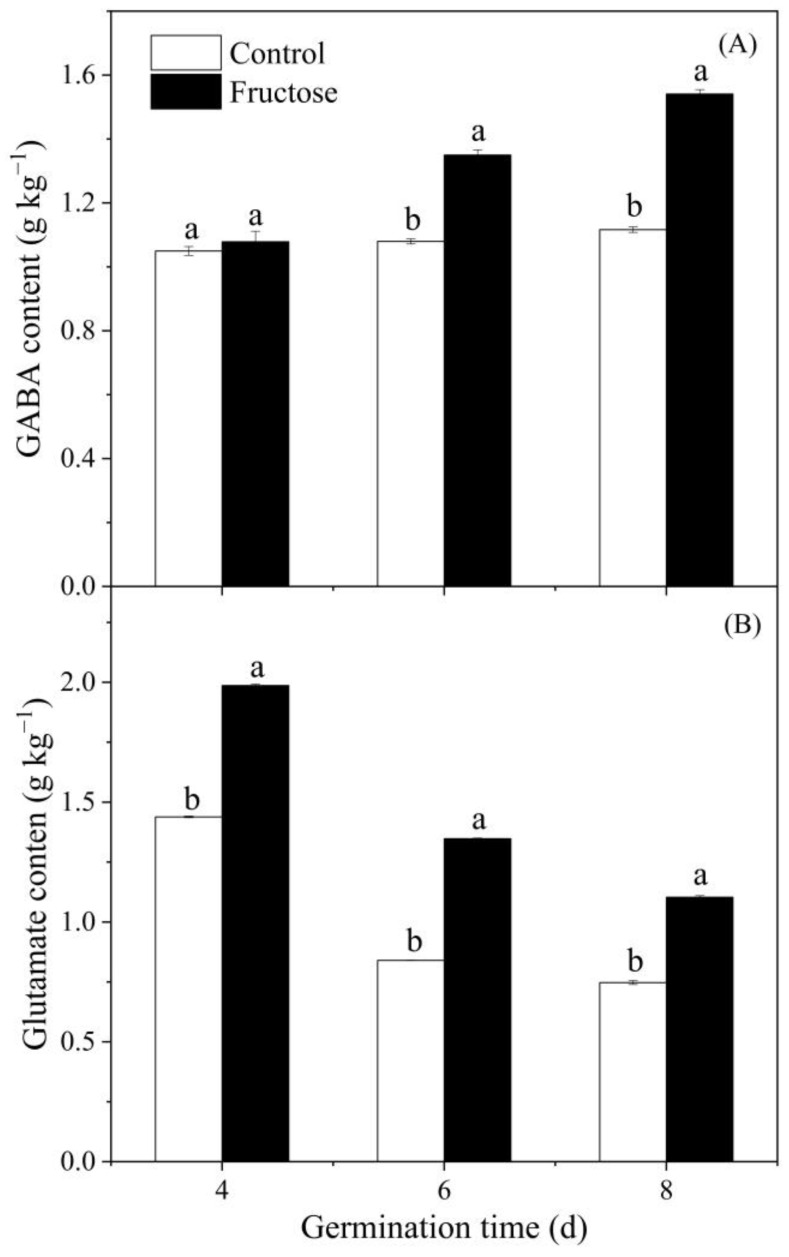
Effect of fructose treatment on GABA (**A**) and glutamate contents (**B**) in broccoli sprouts. Each value represents the mean of three replicates per treatment and time point (mean ± standard error). Data with different letters are signed significantly at *p* < 0.05.

Compared with control sprouts, fructose treatment significantly increased the GABA content (Figure 3A). The glutamate level in both control and fructose-treated groups reduced in sprouts during the germination period. However, the fructose sprouts consistently had more glutamate than the control (Figure 3B).

### 2.3. Effect of Fructose Treatment on ABA, IP_3_ Contents, PI-PLC Activity, and BoPLC Expression in Broccoli Sprouts

**Figure 4:**
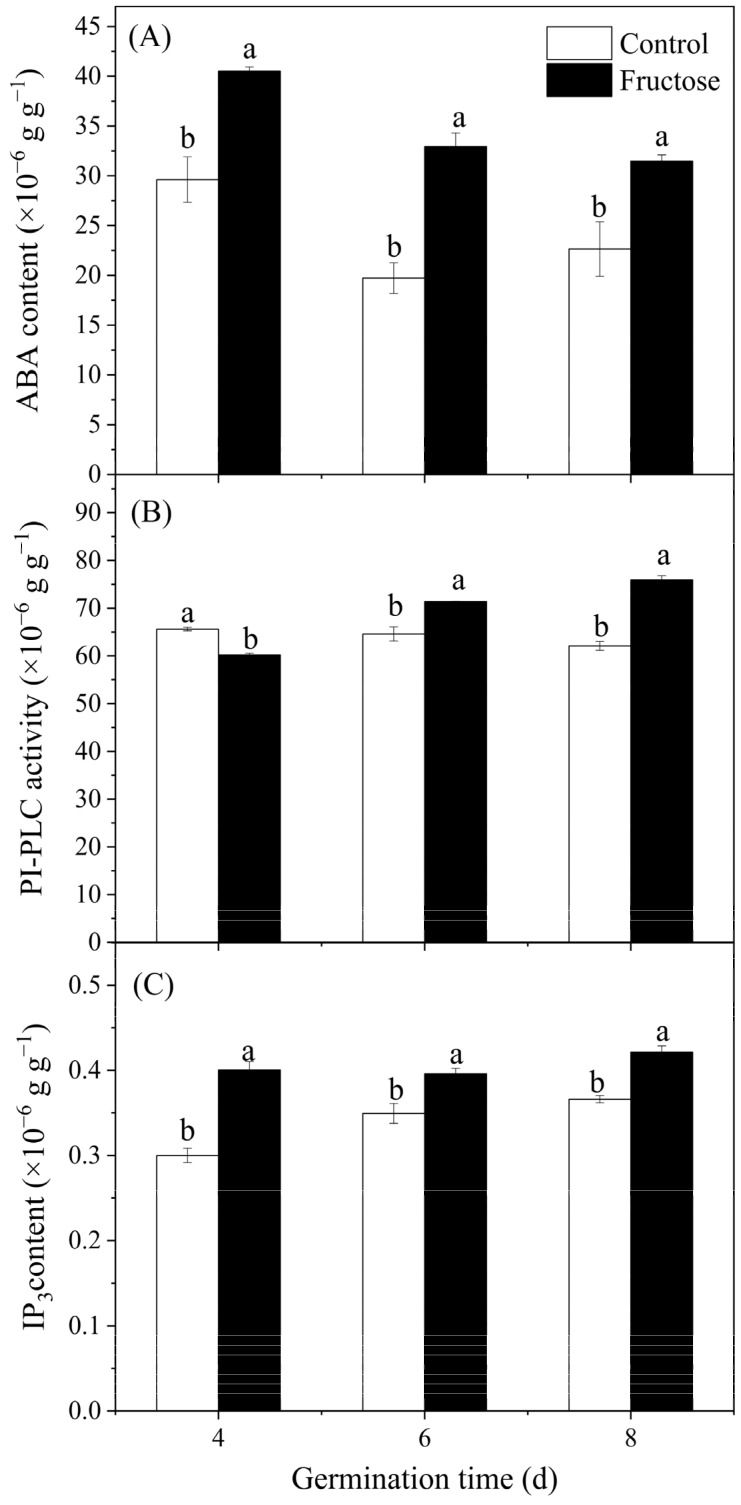
Effect of fructose treatment on ABA content (**A**), PI-PLC activity (**B**), and IP_3_ content (**C**) in broccoli sprouts. Vertical bars represent the ± standard error of the mean. Data with different letters are signed significantly at *p* < 0.05.

The abscisic acid (ABA) content in fructose-treated sprouts significantly increased. The fructose-treated broccoli sprouts maintained a higher ABA concentration in comparison with the controls (Figure 4A). Fructose treatment enhanced the PI-PLC activity, and significantly higher activity was observed in fructose-treated sprouts after 6 and 8 days of germination in comparison with the control sprouts (Figure 4B). When compared to control sprouts, fructose-treated sprouts retained an evidently higher level of IP_3_ during the germination period (Figure 4C).

**Figure 5:**
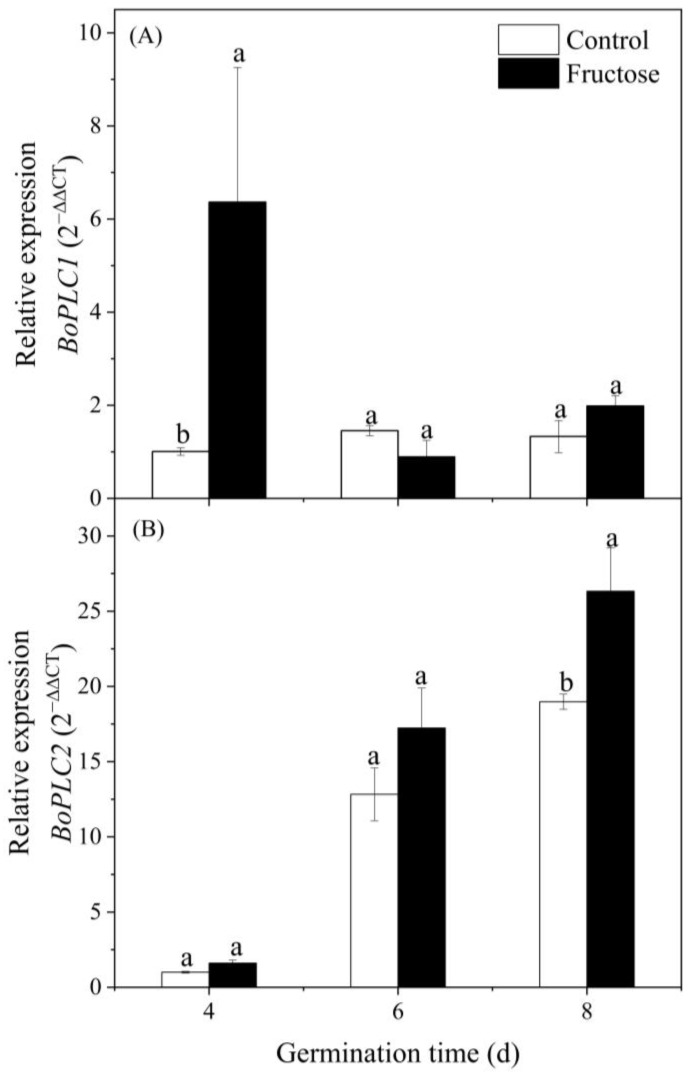
The expression levels of *BoPLC1* (**A**) and *BoPLC2* (**B**) under fructose treatment and control in broccoli sprouts. Vertical bars represent the mean ± standard error. Data with different letters are signed significantly at *p* < 0.05.

As shown in Figure 5A, the expression of *BoPLC1* under the fructose treatment was considerably higher than the control on day 4. In contrast, there was no significant change in *BoPLC1* expression in the control sprouts, and there was no considerable difference between the fructose and control groups on days 6 and 8 (Figure 5A). In contrast, *BoPLC2* expression in both the fructose-treated and control groups showed an increasing trend (Figure 5B), and a clear difference was noticed at day 8. PI-PLC accelerated the conversion of PIP_2_ to IP_3_, and the content of IP_3_ increased significantly under fructose treatment (Figure 4C).

### 2.4. Fructose Treatment Improved Ca^2+^ and CaM Contents in Broccoli Sprouts

**Figure 6:**
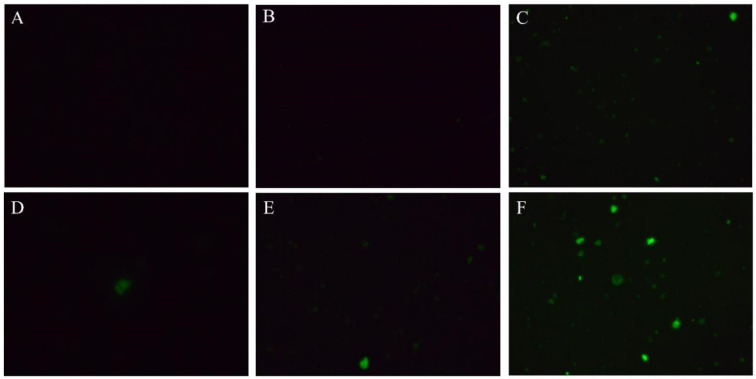
Effect of fructose treatment on Ca^2+^ content in sprouts cells. The fluorescence from stained Fluo-3 AM was observed by Olympus ix81 fluorescence microscope: (**A**) Control sprouts on day 4; (**B**) Control sprouts on day 6; (**C**) Control sprouts on day 8; (**D**) Fructose sprouts on day 4; (**E**) Fructose sprouts on day 6; (**F**) Fructose sprouts on day 8.

**Figure 7:**
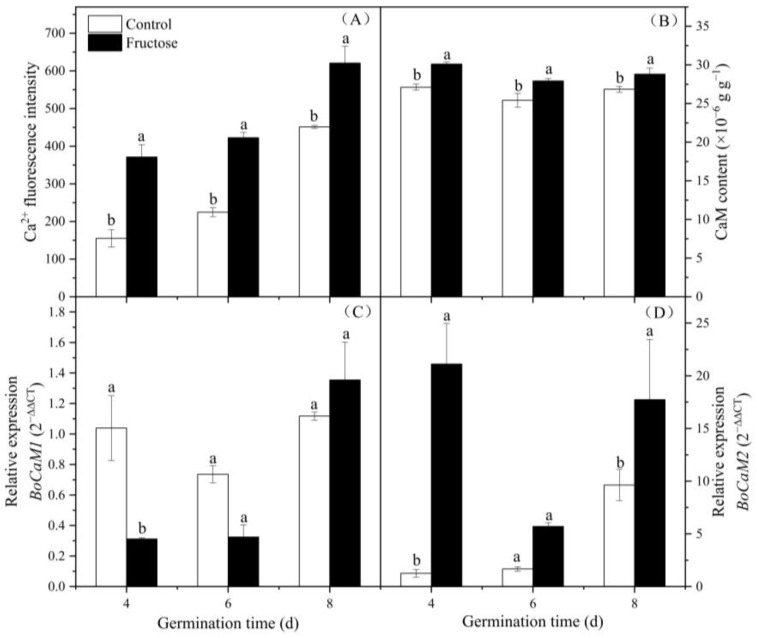
Effect of fructose treatment on Ca^2+^ fluorescence intensity (**A**), CaM content (**B**), and expression levels of *BoCaM1* (**C**) and *BoCaM2* (**D**) in broccoli sprouts. Vertical bars represent the mean ± standard error. Data with different letters are signed significantly at *p* < 0.05.

The sprouts treated with fructose displayed relatively stronger Ca^2+^ fluorescence than that in the controls (Figure 6D–F). Meanwhile, the Ca^2+^ content in all groups gradually increased during the growth process. Fructose treatment markedly increased the Ca^2+^ content, which made the content in the fructose treatment higher than in the control sprouts (Figure 7A). The fructose group retained a stronger CaM content than that in the control (Figure 7B). The expression of *BoCaM1* in the control was stronger than in the fructose-treated sprout on day 4 (Figure 7C). However, the expression of *BoCaM2* in the fructose-treated group was significantly upregulated except on day 6 (Figure 7D).

### 2.5. Effect of Fructose Treatment on the Activities of GAD and GABA-T

**Figure 8:**
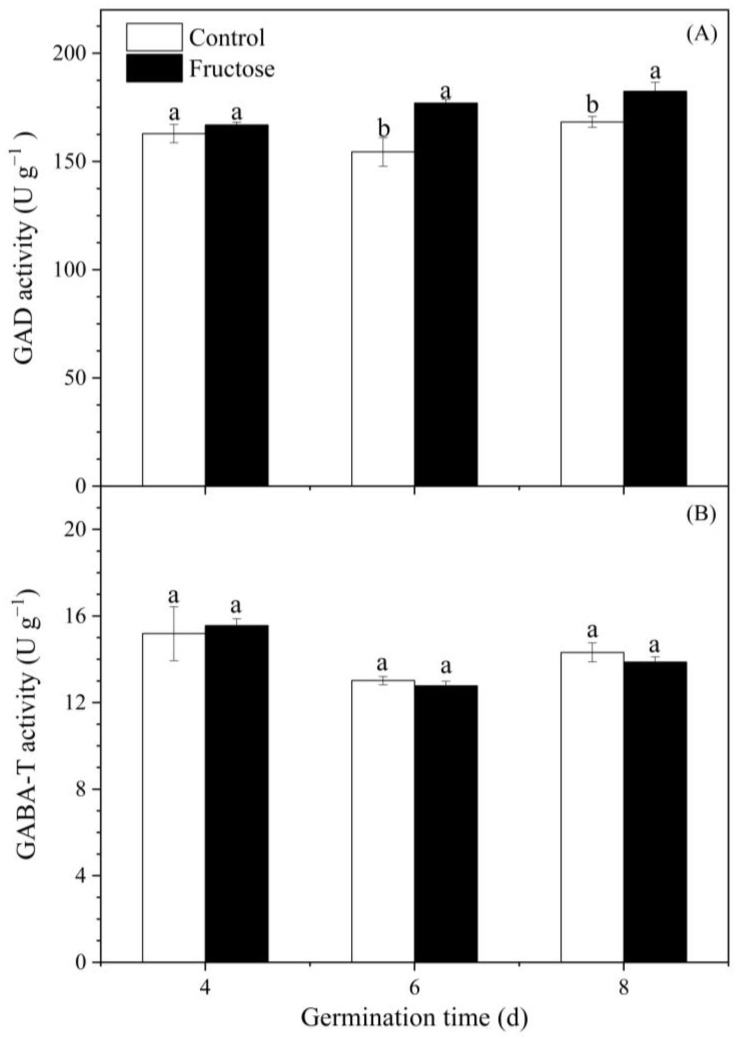
Effect of fructose treatment on GAD (**A**) and GABA-T activity (**B**) in broccoli sprouts. Vertical bars represent the mean ± standard error. Data with different letters are signed significantly at *p* < 0.05.

Fructose-treated broccoli sprouts showed significantly higher GAD activity than the control (Figure 8A). There were no significant differences in GABA-T activity between the fructose-treated sprouts and control sprouts during the germination period (Figure 8B). 

### 2.6. GABA Shunt Metabolism Genes Expression under Fructose Treatment

**Figure 9:**
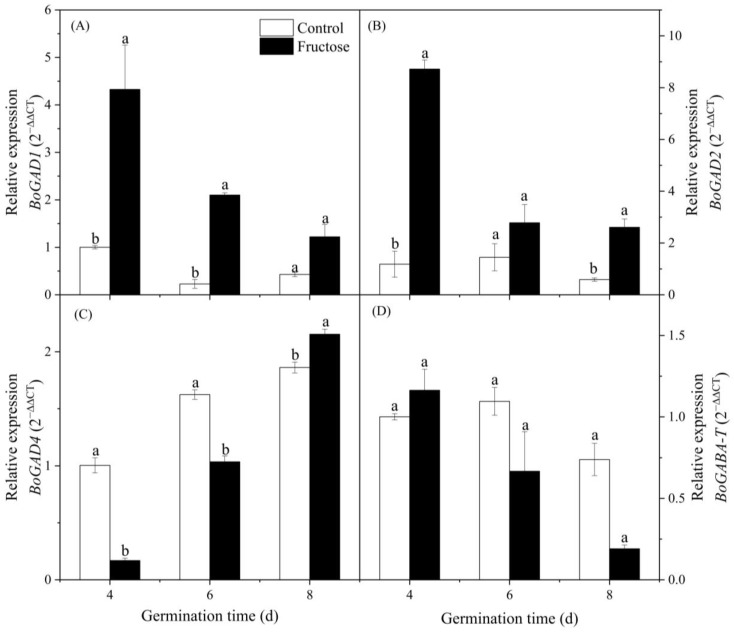
Effect of fructose treatment on the expression levels of *BoGAD1* (**A**), *BoGAD2* (**B**), *BoGAD4* (**C**), and *BoGABA-T* (**D**) in broccoli sprouts. Vertical bars represent the mean ± standard error. Data with different letters are signed significantly at *p* < 0.05.

The expression of *BoGAD1* in control broccoli sprouts tended to decrease during the first 6 days and then increased marginally. The expression in fructose-treated sprouts decreased gradually and presented at a higher level than that in the control (Figure 9A). Similar to *BoGAD1,* the expression of *BoGAD2* showed a higher level than that in the control after 4, 6, and 8 days of germination (Figure 9B). Differently, the expression of *BoGAD4* in all groups tended to increase, and the expression in the fructose-treated sprouts was kept at a lower level than in the control group, except on day 8 (Figure 9C). No significant difference was found in the expression of *BoGABA-T* between the control and fructose-treated sprouts (Figure 9D).

## 3. Discussion

As a precursor of GABA, glutamate content affects GABA enrichment. Exogenous L-glutamic acid resulted in an increase in glutamate content in pears, along with the significant enrichment of GABA [17]. Varieties of quinoa seed with higher glutamate content will have a little more enriched GABA during germination [18]. In our work, fructose treatment significantly improved the glutamate content in broccoli sprouts (Figure 3B). The trend of GABA content was also similar to GAD activity. We inferred that the increase in glutamate content was related to the entry of fructose into the tricarboxylic acid cycle. At the same time, fructose promoted GAD activity (Figure 8A), which induced the GABA accumulation (Figure 3A). This is similar to a previous study in which exogenous mannose boosted GABA accumulation in broccoli sprouts by providing more glutamate and increasing GAD activity [16]. In this study, *BoGAD1* and *BoGAD2* dominated with significantly higher expression levels under fructose treatment. The *PbGAD* expression was also upregulated in fresh-cut pears treated with exogenous CaCl_2_, which resulted in an increase in GAD activity and GABA content [10]. It was reported that the GABA content in germinating soybean significantly increased by enhancing the expression of *GmGAD* and GAD activity [19]. However, the activity of GABA-T and the expression of *BoGABA-T* did not change significantly in the present work (Figure 8B and Figure 9D), indicating that the enrichment of GABA was not due to the inhibition of its catabolism, but to the increase in the substrate (Glu) and activation of GAD activity as well as its gene expression.

It has been found that plant GAD has a CaM-binding domain (CaMBD), and Ca^2+^ binds to CaM to activate GAD when the Ca^2+^ concentration in cytosol increases under external stimulation, while Ca^2+^ or CaM alone do not work [20]. CaM transmits Ca^2+^ signals by sensing changes in intracellular Ca^2+^ concentration [21]. CaM can combine with Ca^2+^ to form Ca^2+^/CaM complexes and interact with downstream calmodulin-binding proteins to play an important role in signal transduction pathways in plant growth regulation and environmental response [22]. Fructose treatment promoted the intracellular Ca^2+^ content (Figure 6 and Figure 7A). This was consistent with earlier studies which reported that the addition of fructose to BY-2 cells increased the intracellular Ca^2+^ content [23]. The Ca^2+^ sensor recognized intracellular Ca^2+^ signals and activated CaM gene expression through a cascade reaction, thereby inducing resistance in plants [24]. Although the expression of *BoCaM1* tended to stabilize and was lower in the fructose-treated group on days 4 and 6, the gene expression of *BoCaM2* showed a steady upregulation with growth (Figure 7D). These results indicated that *BoCaM2* played a dominant role in this process. In this work, the CaM content increased under fructose treatment (Figure 7B), which might stimulate Ca^2+^ content and form the Ca^2+^/CaM complex. The Ca^2+^/CaM complex bound to the CaMBD of GAD significantly increased GAD activity and the GABA content. Exogenous calcium activated GAD activity in shredded carrots and promoted GABA accumulation [9]. CaCl_2_ facilitated GAD activity through extracellular Ca^2+^ influx, stimulating the GABA accumulation in fresh-cut pears [10].

ABA is an important signaling compound which can stimulate GABA synthesis [25]. Under external stimulation, plants will promote ABA synthesis to increase the local concentration of ABA to improve stress tolerance [26]. In this study, fructose treatment exhibited an inhibitory effect on sprout stem length and enhanced the ABA content (Figure 1 and Figure 4A). The findings were in keeping with the reduction in the length of broccoli sprouts treated with mannose [16,27]. It is suggested that a 60 mmol L^−1^ concentration of fructose is detrimental to the growth conditions of broccoli sprouts. It has been reported that ABA treatment resulted in a significant increase in the levels of IP_3_ in the guard cell protoplasts of Vicia faba [28].

IP_3_ is the product obtained from the decomposition of PIP_2_ by PI-PLC, and IP_3_ is released from the cell membrane into the cytoplasm to bind to the designated receptors [29]. In this study, fructose treatment increased the activity of PI-PLC (Figure 4B) and upregulated the expressions of *BoPLC1* and *BoPLC2* (Figure 5). The amount of IP_3_ significantly increased in broccoli shoots (Figure 4C). IP_3_ can activate Ca^2+^ channels in the inner membrane of plant cells [30]. The increase in IP_3_ allowed more Ca^2+^ to enter the sprouts and Ca^2+^ fluorescence intensity in broccoli sprouts (Figure 6 and Figure 7A). Our data were agreement with the findings that *Arabidopsis* regulated the increase in intracellular Ca^2+^ by increasing PI-PLC activity and IP_3_ content, which was used to mitigate the effects of environmental stresses [31]. In soybean sprouts, UV-B induced the accumulation of IP_3_ and improved the contents of Ca^2+^ and GABA [32]. Fructose could be considered as a potential option to improve the nutritional value in broccoli sprouts. Moreover, the detailed mechanism regulating GABA accumulation requires further exploration.

## 4. Materials and Methods

### 4.1. Seed and Cultivation Conditions

Broccoli seeds (*Brassica oleracea* var. italica cv. Yanxiu) were bought from Sakata Seed (Japan) Corporation. Seeds were disinfected with 2 % sodium hypochlorite for a period of 1 min then emptied and soaked with distilled water at 30 °C for 4 h. The concentration of 60 mmol L^−1^ fructose was selected according to our previous study (data not shown). Seeds were moved to the bean sprout growing machine (DYJ-S6108, Guangdong, China) full of distilled water (control) and 60 mmol L^−1^ fructose. Next, they were germinated with a cycle of 14 h of light and 10 h of darkness. The sprouts were harvested on days 4, 6, and 8 after seeding. All samples were taken and kept frozen in liquid nitrogen at −80 °C for analysis at a later date.

### 4.2. Sprouts Stem Length and Fresh Weight Measurement

Vernier calipers were used to measure the length of the broccoli sprouts’ stem. The fresh weight of the broccoli sprouts was measured after cut roots. Each group was tested 30 times using a parallel test.

### 4.3. Determination of GABA Content

GABA content was extracted according to Al-Quraan et al. [33] and Hu et al. [34] with the following modifications. A total of 0.2 g of sprouts was obtained using extraction with methanol (0.2 mL) and 70 mmol L^−1^ LaCl_3_ (1 mL) on ice. The mixture was centrifuged for 5 min at 8000× *g*. Then, a total of 0.8 mL supernatant and 0.2 mL of 1 mol L^−1^ KOH was mixed and centrifuged for 5 min at 8000× *g*. 

The supernatant (2.0 mL) was added to a tube containing 0.3 mL of phosphate buffer (pH 9.0), 1 mL of 6% (*w/v*) phenol, and 0.5 mL of 5% (*v/v*) NaClO, and the sample was placed in boiling water for 10 min and ice for 5 min, respectively. The absorbance was determined at 654 nm.

### 4.4. Analysis of Glutamate Content

Frozen sprouts (0.2 g) were dissolved in 0.2 mL methanol for 10 min and 1 mL of 70 mmol L^−1^ lanthanum chloride was added. The mixture was centrifuged at 8000× *g* for 5 min. The mixture of 0.8 mL supernatant and 0.2 mL of 1 mol L^−1^ KOH was centrifuged at 8000× *g* for 5 min. Then, the glutamate content was then determined according to Xie et al. [16].

### 4.5. Abscisic Acid (ABA) Content Determination

The frozen sample (0.2 g) was homogenized in 1 mL of normal saline and completely transferred to a centrifuge tube. Centrifugation took place at 4 °C at a speed of 3000× *g* for 20 min. The content was assayed by the plant ABA ELISA kit (Jiangsu, China).

### 4.6. Determination of Phosphatidylinositol-Specific Phospholipase C (PI-PLC) Activity

The activity of PI-PLC was detected using a plant PI-PLC ELISA kit (Jiangsu, China). The frozen sample was grinded in phosphate buffered saline (PBS) solution (pH 7.4) and completely transferred to a centrifuge tube. Centrifugation took place at 4 °C at a speed of 3000× *g* for 20 min. The activity of PI-PLC in the supernatant was determined by following the manufacturer’s instructions.

### 4.7. Inositol Trisphosphate (IP_3_) Content Assay

The IP_3_ content was detected using a plant IP_3_ ELISA kit (Jiangsu, China). A total of 0.2 g of sprouts was homogenized with 1 mL of normal saline and centrifuged at 4 °C with a speed of 3000× *g* for 20 min. 

### 4.8. Analysis of Ca^2+^ and CaM Contents

The cell culture was slightly modified as described by Bethke and Jones [35]. Broccoli sprouts were collected at 4, 6, and 8 d incubation for cell culture. The samples were chopped and cultured in liquid plant medium (containing 0.02 g 2,4- dichlorophenoxyacetic acid, 0.02 g 6-benzylaminopurine, 0.6 g pectinase) at pH 5.8. The 100 mL conical flasks with the medium were incubated at 25 °C for 24 h with a shaker shaking at 110× *g* and protected from light.

Ca^2+^ content was analyzed using a Fluo-3 AM fluorescent probe according to Li et al. [36]. The collected cells were washed three times with pre-cooled PBS. The cells were stained with 5 µmol L^−1^ Fluo-3 AM dye for 20 min at 37 °C and washed three times with HBSS buffer. Finally, the cells were suspended in 1 mL HBSS buffer and incubated at 37 °C for 15 min. The relative fluorescence intensity of cytosolic Ca^2+^ in budding cells loaded with Fluo-3 AM was then measured.

CaM content was determined according to Chi et al. [10]. The frozen sample was homogenized with 1 mL of Tris−HCl buffer (pH 7.5) and centrifuged at 4000× *g* for 10 min. CaM content in the supernatant was determined following the manufacturer’s instructions.

### 4.9. GAD and GABA-T Activities Assay

Broccoli sprouts (0.2 g) were homogenized with 60 mmol L^−1^ phosphoric phosphate buffer (pH 5.8), which contained 2 mmol L^−1^ EDTA, 0.5 mmol L^−1^ pyridoxal phosphate, and 5 mmol L^−1^ β-mercaptoethanol on ice. Centrifugation took place at 4 °C at a speed of 10,000× *g* for 20 min. The mixture was analyzed for GABA content according to Hu et al. [34]. 

For assay of GABA-T activity, the frozen sprouts were homogenized in 1 mL 100 mmol L^−1^ Tris-Cl (pH 9.1 including 10% glycerol, 1 mM dithiothreitol, 0.5 mM pyridoxal phosphate, 5 mM EDTA, and 1 mM phenylmethylsulfonylfluoride). The mixture centrifuged at 4 °C with a speed of 10,000× *g* for 20 min. GABA-T activity was detected according to Wang et al. [9].

### 4.10. Gene Expression Analysis

Total RNA from broccoli sprouts was extracted using a plant Total RNA isolation kit (Vazyme). cDNA was synthesized using the R223-01 HiScript^®^ ii Q RT SuperMix for qPCR (+ gDNA WIper) kit provided by Vazyme. The sequence-specific primers were listed in Table 1. The reaction was performed using the Q712-02 Cham QTM SYBR^®^ qPCR Master Mix kit and ABI Prism 7500 (Vazyme) Fast qPCR instrument provided by Vazyme. The synthesized cDNA was used as the template for qPCR amplification. After qPCR was completed, the relative gene expression was calculated by 2^−ΔΔCT^ method.

### 4.11. Statistical Analysis

All data were analyzed using analysis of variance (ANOVA model one-way) and the SPSS (SPSS Inc., Chicago, IL, USA). The values were expressed as means with their standard error (SE) for all results. Mean separations were analyzed using Duncan’s multiple range tests at a significance level of 0.05.

## 5. Conclusions

Fructose treatment was effective for stimulating GABA accumulation in broccoli sprouts via the activation of GAD activity and expressions of *BoGAD1* and *BoGAD2.* Meanwhile, it acted as an external stimulus, inhibiting the broccoli sprouts stem length and enhancing the ABA content. Fructose promoted the activity of PI-PLC and IP_3_ production and induced Ca^2+^ accumulation. Ca^2+^ can activate GAD activity and its gene expression used for GAD biosynthesis and contributes to GABA enrichment by enhancing the conversion of Glu. Fructose could be considered as a potential option with which to improve the nutritional value in broccoli sprouts.

## Figures and Tables

**Table 1:** PCR primer sequences and PCR conditions used for the expression analysis of GAD, GABA-T, PLC, and CaM genes.

Gene	Accession No.	Primer	Primer Sequence (5′-3′)
***18S***	AF513990	18S-F	CGAGACCTCAGCCTGCTAACT
		18S-R	CAGAACATCTAAGGGCATCACA
***BoGAD1***	LOC106329129	GAD1-F	GGTGACGGTGAAGAAAACCG
		GAD1-R	CCTTCAAATCTCCGAATTAGTGC
***BoGAD2***	LOC106300456	GAD2-F	ATCTCGCTATGTCCGCACTG
		GAD2-R	TCTGGAGCTCGGTAGTGACA
***BoGAD4***	LOC106325355	GAD4-F	AGGGTTCACGCTAAGATGGC
		GAD4-R	CCATGGGAGAAAGGGCTTCA
***BoGABA-T***	LOC106391515	GABA-T-F	TTGATTCTGGGAACTGAG
		GABA-T-R	TGAGATAATAAGCGGTGG
***BoPLC1***	LOC106322918	PLC1-F	CGTGGACCCGATTTAGTG
		PLC1-R	TGCATATTGAAGGCAACC
***BoPLC2***	LOC106312862	PLC2-F	CCCGATTTCTACGCAAGGGT
		PLC2-R	ACGCAATGGGAACTCGAACT
***BoCaM1***	LOC106317776	CaM1-F	CTCTTCGACAAGGATGGTGAC
		CaM1-R	GGTTTTGCCCTAGAGACCTCA
***BoCaM2***	LOC106327158	CaM2-F	AGTTCCTGAACCTGATGGCG
		CaM2-R	TCAGCTTCTCCCCGAGGTTA

## Data Availability

Data not available due to ethical or privacy restrictions.
